# Analysis and strategy research on quality of nursing work life

**DOI:** 10.1097/MD.0000000000019172

**Published:** 2020-02-07

**Authors:** Lei Wang, Xuerui Wang, Shuang Liu, Binquan Wang

**Affiliations:** a College of Nursing, Shanxi Medical University; b Administration Department, The First Hospital of Shanxi Medical University, Taiyuan City, Shanxi Province, China.

**Keywords:** nursing, quality of working life

## Abstract

Quality of Working Life (QWL) was developed in 1970s as a new theory on a basis of social-technical system theory. In 2004, Brooks considered that quality of nursing work life is a degree to which the registered nurses are able to satisfy important personal needs through their experiences in their work organization's goal. Quality of Nursing Work Life plays an important role in nursing management.

The purpose of the project was to identify factors associated with nursing work life quality.

A convenience sample of 3498 nurses from five tertiary general hospitals in Shanxi, Shandong, and Liaoning provinces in China was surveyed regarding quality of work life, working conditions, stress at work, general being, and job and career satisfaction (JCS).

The mean overall quality of work life score was found to be 3.40 ± 0.61 (on a scale of 1–5, with 5 being the highest), while the working conditions and stress at work received lower scores. The general well-being of females (3.49 ± 0.74) was higher than that of males (3.35 ± 0.87). We also found a statistically significant difference of JCS of different department groups (*P* = .004).

The quality of working life of nurses was found to be in the middle range, with room for improvement. Nurse managers have an opportunity to implement measures to improve the quality of working life for nurses in China.

## 1 Introduction

At present, the intense nursing work and the serious lack of nursing staff have become a high-profile international problem, which is closely related to the stability of the nursing industry, the quality of nursing services and the satisfaction of nurses and patients.^[1]^ Improving the quality of work and life of nurses, ensuring organizational stability, and reducing turnover rate are one of the most challenging issues at present, and they are also an urgent problem for medical administrators. Professor Dr. Wayne Kashaw of American Psychology believes that managers should pay attention to improving the quality of life in the work process of employees when studying the issue of production efficiency, and puts forward the “Quality of Working Life (QWL),” which means that the physical and psychological sensations of the staffs produced at work. This concept is designed to help organizations attract and retain talented employees to ensure high-quality performance, and was first developed and widely used in the field of industrial labor psychology^[2]^

In order to do a specific assessment of the special occupational group of nurses, nursing-related managers have conducted deep research on the quality of professional life of the nurses in recent years. In 2004, Brooks et al^[3]^ proposed that QWL is the extent to which nurses meet their important individual needs through experience while completing organizational goals. Studies have shown that occupational stress and emotional exhaustion are positively correlated. The psychosocial environment is particularly important for nurses’ mental health. Especially for nurses in traditional countries, they are the most likely nurses to leave their jobs in the case of serious imbalances between pay and return.^[4]^

In China, nurses account for a large proportion of the entire health care system and are the most connected to the patients. The characteristics of nursing work include four points^[5]^:

1. Nurses contain many positions and their work is complex, diverse, and professional;
2. The severity of the illness is impermanent so that many departments need 24 h monitoring such as emergency department and ICU;
3. Knowledge is updated quickly, new instruments and technologies are often put into use in operating rooms and intensive care units;
4. Chinese medical system is implemented with tertiary medical coverage, with community health service centers, villages health clinics, township hospitals, county-level hospitals, and municipal-level general hospitals, and has different levels of nurses.

Studies have shown that,^[6]^ occupational stress and emotional exhaustion are positively correlated, and the psychological environment is particularly important for nurses’ mental health. In 2010, the National Nursing work Conference launched the “China Quality Care Service Demonstration Project” with the theme of “tamping basic nursing and providing satisfactory services.” In the Project, the National Health and Family Planning Commission clearly stated that hospitals should care for nurse's quality of life and physical and mental health, and protect nurse's legal rights. However, in a complex and high-intensity work environment, the lack of social support and the long-term high-paying and low-reward psychological environment affect the quality of life and physical and mental health of nurses, and which indirectly affect the safety of patients.^[7]^ The quality of professional life of nurses is closely related to their work efficiency, job satisfaction, quality of care and hospital development, and has a certain role in promoting the reduction of nurse turnover rate and stabilizing the nurse team. Paying attention to and improving the overall quality of life of Chinese nurses is not only conducive to promoting the physical and mental health of nurses, but also to improving the enthusiasm of nurses, which is more conducive to stabilizing the nursing team and ensuring the safety of patients. Therefore, this study investigated the current status of work-related quality of clinical nurses in five tertiary general hospital, in order to provide a reference for the stable development of the nursing team.

## 2 Methods

### 2.1 Subjects

The study was conducted from September 2017 to October 2017 in five hospitals in China. (First Hospital of China Medical University is a 2249 beds, large-scale tertiary general hospital. Cancer Hospital of China Medical University is a 2330 beds, tertiary care cancer's hospital. Qilu hospital of Shandong University is a 4500 beds, large-scale tertiary general hospital. Shandong Provincial Hospital is a 3500 beds, large-scale tertiary general hospital. And First Hospital of Shanxi Medical University is a 1500 beds, tertiary general hospital.) Survey participants worked in a variety of departments, including internal medicine, surgery, gynecology, pediatrics, outpatient, emergency, and operating room. Participant inclusion criteria were: registered nurses who have a nurse practitioner certificate and clinic nurses. All the nurses volunteered to participate in the survey and signed an informed consent form. This study was approved by the Regional Ethical Review Board (Reg. No. 2018/K-K002).

### 2.2 Survey instrument

#### 2.2.1 The general questionnaire of nurses

The General Questionnaire of Nurses was designed by members of the research team. According to the purpose of the study and similar studies, 18 questions were designed, including the following variables: gender, age, years of nursing work, department, title, education, job nature, average monthly salary, marital status, frequency of night shift monthly, pregnancy status, breast feeding status, numbers of children in need of care, age of youngest child, number(s) of the elderly in the home and degree of dependence, presence of sleep disorder, chronic disease, and frequency of required overtime work for the nurse.

#### 2.2.2 Work-related quality of life scale (WRQOL-2)

WRQOL-2 was adjusted by Chinese scholars Shao Ya and Liao Shaoling.^[8]^ This tool includes 7 dimension of the working conditions scale (WCS), stress at work (SAW), control at work (CAW), home–work interface (HWI), employment evaluation of nurse (EEN), general well-being (GWB), and job and career satisfaction (JCS). The 5 point scale scoring method was used to assess level of agreement, where 1 means “totally disagree” and 5 means “strongly agree.” Higher score signify higher quality of nursing work life. The sample size of the pilot test was based on the principle of 3 to 5 times the number of items of the sub-scale, so 100 nurses were selected as the subjects of the pilot test.^[9]^ In WRQOL-2, the α reliability (Cronbach's alpha) is 0.958 and split-half reliability is 0.936, which reflects the high Internal consistency within test items.

### 2.3 Methods of data collection

The preparation of questionnaire was completed via sojump.com. After providing informed consent, nurses were given instructions to read and answer each question. A total of 5000 questionnaires were distributed, and 3498 valid questionnaires were recovered, for a response rate of 69.96%.

### 2.4 Statistical treatment

SPSS20.0 statistical software was used to calculate descriptive statistics, one-way analysis of variance, *t* test and variance analysis are adopted for additional analysis. Differences were identified where *P* < .05.

## 3 Results

### 3.1 Analysis of general information

Respondents included 204 male nurses (5.83%) and 3294 females (94.17%). The age range from 20 years old to 55 years old; 70.53% were married, and the majority 84.36% had 3 or more years of work experience. 6.17% of the nurses were pregnant, and 11.29% were breastfeeding. A small percentage 7.58% had children younger than 2 years of age, while about one half 51.83% had responsibilities to care for the elderly; nurses with bachelor or above are 86.71%; nurses in system of engagement are 73.07%; nurses with 6000 or above monthly income are 53.92%. Table 1 provides an overview of the sample characteristics.

**Table 1:**
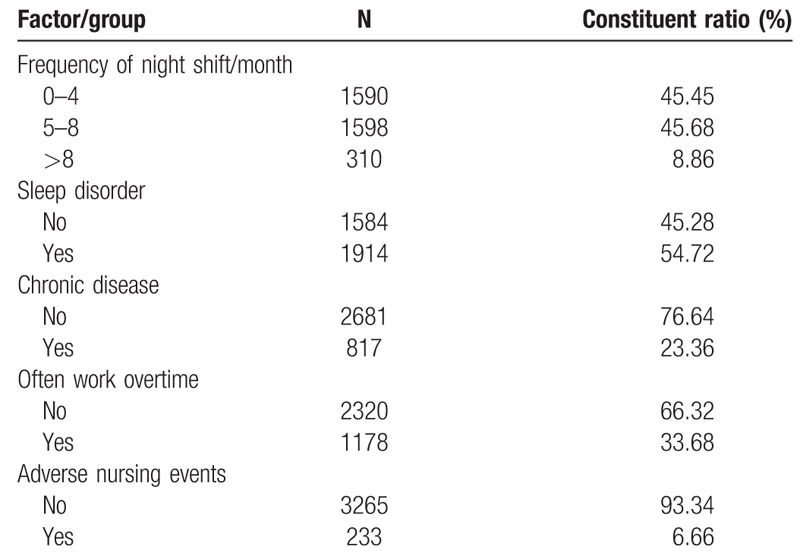
General situation of nurses (N = 3489).

### 3.2 Quality of nursing work life

The average score of for the 7 dimensions in quality of nursing work life was (3.40 ± 0.61). The highest scores were for JCS while the lowest scores were for SAW. Table 2 provides a summary of the quality rankings for each key area.

**Table 2:**
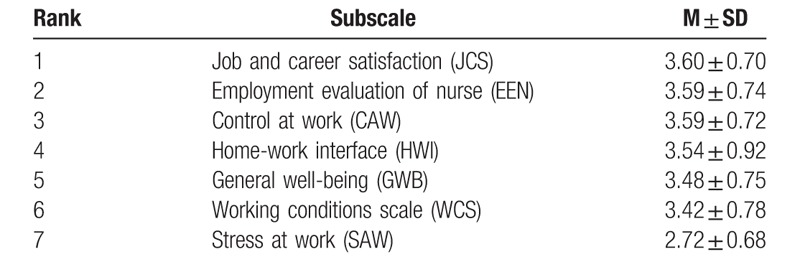
Score for every scales of quality of nursing work life (M ± SD).

### 3.3 Score comparison of quality of nursing work life between nurses with different characteristics

The following factors were found to correlate with reported quality of work life: age, title, education, job nature, sleep disorder, and adverse nursing events or not. See Table 3.

**Table 3:**
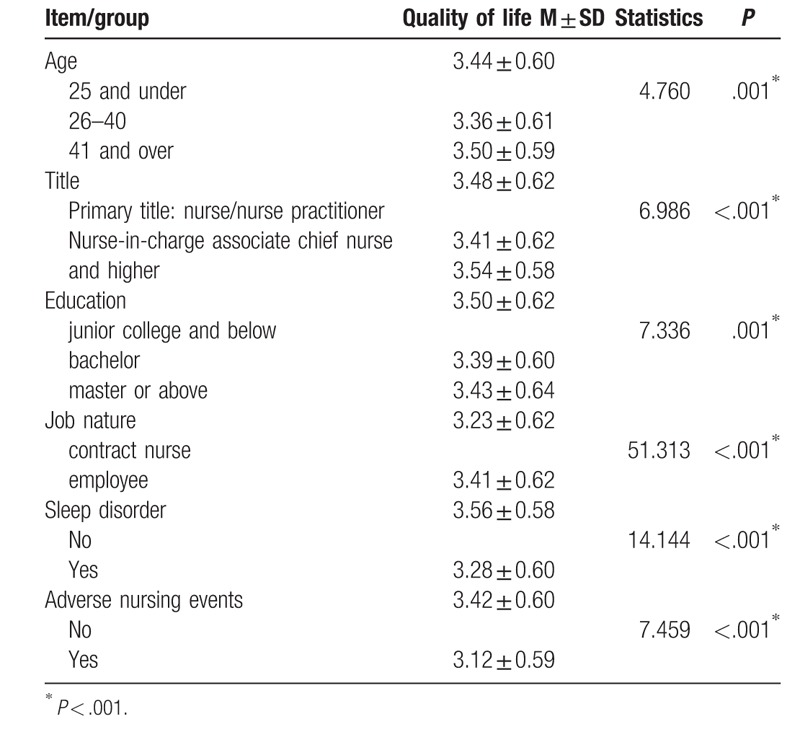
Factors that correlate with quality of nursing work life.

## 4 Discussion:

### 4.1 Status of nurses’ work and life quality

At present, the definition of QWL at home and abroad has not been unified. Most foreign scholars believe that QWL is the subjective feeling of individuals for work, organization and managers. With the development of nursing disciplines, the emphasis on clinical nurses QWL is more and more obvious at home and abroad. The level of clinical nurse QWL determines the efficiency of work and the quality of care.^[10,11]^ The results of this study show that the QWL scores of clinical nurses are significantly different in different ages, titles, education, work nature, presence, or absence of sleep disorders, and the occurrence of adverse events. This is roughly the same as the results of many domestic scholars. Li Weihua^[12]^ surveyed 600 nurses who were officially employed in a tertiary general hospital in Guangzhou. Most of the nurses were between 20 and 30 years old, 70.2% of the nurses were employed nurses. 66.3% of the 600 subjects considered that the promotion opportunities in the work are unfair, so the promotion success has a significant effect on the quality of work and life. Han Xue et al^[13]^ showed that the employment contract nurses are young, low education, low professional titles, low income, poor welfare, and less development opportunities, the score is significantly lower than the nurses in the compilation, which indicated different identities, treatments, and job stability. The work pressure of employed nurses is high, but no attention is given, resulting in no improvement in their quality of work and life. Luo Hongchao^[14]^ showed that the quality of work and life of nurses varies in different ages, working years, titles, departments, late night shifts, marriage, education, professional titles, personnel and labor relations, monthly income, and organizational support affects the quality of work and life of nurses. Psychological empowerment is a mediator variable that affects the quality of work and life of nurses. In summary, a variety of factors have seriously affected the quality of nurses’ quality of work and life.

With the rapid development of science and technology and medical care, people's demand for health is getting stronger and stronger, and the requirements for nursing staff are getting higher and higher, which makes the pressure on nurses increase day by day, which not only affects the quality of nursing work, but also affected the quality of life of nursing staff. Among the subjects surveyed by the Institute, different nurses assumed multiple social roles. The total quality of life related to nurses’ work-related quality (3.40 ± 0.61) was at a medium-high level. Comparing the average scores of each dimension showed that the work stress score was the lowest. The reason may be derived from the following aspects. At present, the tertiary general hospital have a large number of patients, the disease is heavy and complicated, the application of new technologies and new instruments is high, the social expectations are high, the human resources allocation of nurses is insufficient, and the frequency of overtime and night shifts is often high,^[15]^ so that the task of nurses is heavy and cumbersome and life is irregular. In addition, hospital management is strict, nurses have high requirements and frequent assessment, so that nursing staff must not only have a solid theoretical knowledge base, skilled operation skills, but also good communication skills. The ability to continuously learn new knowledge, new skills, and conduct research and guidance will result in a decline in the quality of life of nurses.^[16–20]^

### 4.2 Analysis of factors affecting the quality of work and life of nurses

#### 4.2.1 Age

There is a significant difference in the QWL of nurses of different ages, which suggested that the nursing manager can satisfy the work needs of nurses of different ages on the basis of investigating the QWL of nurses, improve the satisfaction of nurses and build a stable and harmonious nursing team.

#### 4.2.2 Intermediate title and education

The QWL score of nurses with medium professional titles and academic qualifications is lower than other subgroups. The main reason is that the promotion of nurses has a small space and high requirements, which suggested that care managers should especially help the middle titles nurses. According to the nurse's title, ability and education, managers can optimize the nursing human resources, promote the rationalization of personnel distribution, plan the post level management, do a good job skills improvement plan, and formulate long-term career planning.

#### 4.2.3 Employed nurses

The QWL total score of the employed nurses is lower than that of the nurses in the compilation. Although most of tertiary general hospitals implements the Mechanism of Equal pay for Equal work to promote the equivalence of workload and salary, the phenomenon that the quality of life of the employed nurses is lower than the nurses in the compilation has not disappear with the effective development of equal pay for equal work. Therefore, the hospital administrators should pay more attention to the employed nurses, increase their sense of belonging, and build a harmonious and loving working atmosphere while improving the nurses’ salary and welfare bonuses.

#### 4.2.4 Nurses with sleep disorders and adverse events

The QWL scores of nurses with sleep disorders and adverse care events were lower than those without sleep disorders and no adverse care events. The lack of nursing resources and the high pressure increased the incidence of adverse events in nursing. Hospital managers should be aware of the correlation between nurses’ high-intensity work stress, sleep disorders caused by shift work and quality of life. Some methods can be adopted, such as improving department environment, adopting flexible scheduling mechanism to arrange nurses’ working time reasonably, expanding nursing team, and conducting psychological evaluation and intervention on nurses on a regular basis.

### 4.3 Suggestions on improving the quality of work and life of nurses

The quality of work and life has become an important part of human resource management. Nurses are an important part of hospitals. The quality of work and life of nurses is the driving force for nursing staff to practice and improve the quality of nursing. Improving the quality of nurses’ work and life can improve the job satisfaction of nurses and promote organizational goals are completed efficiently.

The study shows that nursing managers can improve the quality of work and life by improving the psychological flexibility of nurses and assigning tasks according to seniority and ability. Some researchers^[20]^ proposed that the application of two-factor theory can mobilize the autonomy and enthusiasm of nurses and improve the level of psychological flexibility. The theory divides the motivation-related factors that make people work hard into two categories: incentives and health factors. Domestic scholars^[21]^ propose that improving the quality of work and life of emergency nurses can be based on incentive factors, and managers can arrange and assign nurses according to the actual needs and motivations of nursing staff. Starting from the health care factors, according to the characteristics of high-age nurses with rich work experience, strong clinical work ability and good coordination ability, the work of high, medium and low-grade nurses is reasonably arranged to give play to the advantages of different years of nurses. For example, for low-grade nurses (work <5 years), most of them are unmarried, and the burden of family is lighter; they are full of energy and lack of clinical experience; they have more time, but they do not have enough salary to effectively meet their own needs. For such nurses, the night shift frequency and shift schedule can be appropriately increased to cultivate their clinical skills and resilience, as well as increase salary. For high-grade nurses (work >10 years), most of them have been married and have children so that their energy and physical function decline, so they can reduce their night shifts, increase guidance and teaching work, and increase rest time after night shifts.

In addition, the ability of nursing managers and support for nursing work is an important factor in the job satisfaction of nurses.^[22]^ The stronger the manager's ability and the more supportive the nursing work, the more the nurses will have a strong sense of trust, security and belonging, which can stimulate nurses’ work enthusiasm and dedication, mobilize the positive emotions and improve job satisfaction. Therefore, nursing managers should continuously improve their leadership level and ability, advocate nurse-centered care of subordinate leadership style and democratic participatory leadership style; provide nurses with good material and emotional support within their own capabilities; give the nurses affirmation and recognition as much as possible, and let the nurses feel that “leaders are always by their side.” This has a very positive effect on improving the job satisfaction of nurses, helps to fully mobilize the enthusiasm and initiative of nurses, and improves the psychological flexibility and enthusiasm of nurses.

## 5 Implications for practice

Our research provides new evidence that age, job title, education, job nature, sleep disorders, and adverse care events are factors that influence the quality of work and life of nurses. Hospital managers can carry out scientific management of nurses according to this result, reform the environment, scheduling, and system according to the different factors affecting the QWL of nurses, and carry on the psychological evaluation and the appropriate psychological behavior intervention to the nurses, so as to improve the QWL of nurses.

## 6 Limitations

The study was conducted with the RNs nurses working at tertiary-level hospitals. The tertiary hospitals are the hospitals where the maximum supplies of the resources were available. Other hospitals like general hospitals and district level hospitals received less supplies and resources for patient care. The findings of the study might not be generalized in all hospital settings. The data were collected through a self-report questionnaire, which may not reflect the real situation. Besides, the study whereby is 95% females and 5% males, which does not seem to fully reflect the impact of different genders.

## 7 Conclusion

All in all, because nursing work is important to hospital administration, health administrative department should formulate the relevant regulations and think highly of the importance of nurses to enhance the social position of them. Hospital managers need to carry out the human-based management, hold QWL meetings regularly, attach importance to labor value, promote the benefits of contract nurses, make fair plan to distribute performance bonus, select and appoint fairly, and ensure equal promotion mechanism. Nurse managers should respect the views of nurses, place emphasis on the demands of nurses, add more nurses if necessary, and improve the work environment of nurses. Moreover, nurses ought to keep positive working attitude and develop good living habits to enhance the individual and occupational quality and establish a harmonious interpersonal relationship.

With the development of Chinese Health Care, it is a trend to pay more attention to the quality of nursing work life, but there are more and more factors affecting the QWL of nurses. Therefore, managers need to evaluate nurses regularly and carry out targeted scientific management and effective intervention according to their influencing factors, so as to improve the QWL of nurses.

## Acknowledgments

The authors are grateful for the generous participation of the nurse in this research. They sincerely thank the assistance of the nursing staff of the five third-grade hospitals in Shanxi, Liaoning, and Shandong provinces.

## Author contributions

**Data curation:** Xuerui Wang, Shuang Liu.

**Formal analysis:** Xuerui Wang, Shuang Liu.

**Writing – original draft:** Lei Wang.

**Writing – review & editing:** Binquan Wang.

Lei Wang orcid: 0000-0001-5048-1680.
